# Secondary outcomes of a school-based universal resiliency training for adolescents: a cluster randomized controlled trial

**DOI:** 10.1186/1471-2458-14-1171

**Published:** 2014-11-18

**Authors:** Yuli R Tak, Marloes Kleinjan, Anna Lichtwarck-Aschoff, Rutger CME Engels

**Affiliations:** Behavioural Science Institute, Radboud University Nijmegen, P.O. Box 9104, 6500 HE Nijmegen, The Netherlands; Trimbos-Institute, P.O. Box 725, 3500 AS Utrecht, The Netherlands

**Keywords:** Depressive symptoms, Depression, Resiliency, Universal prevention, Adolescence

## Abstract

**Background:**

The study investigated the long-term effectiveness of the adolescent cognitive behavioral resiliency training Op Volle Kracht (OVK) on the secondary outcomes: anxiety symptoms, hopelessness, happiness, life satisfaction, optimism, coping, self-efficacy, and school functioning. In addition, the study analyzed whether the secondary outcomes moderated the intervention effect on depressive symptoms.

**Methods:**

A two-condition (intervention and control) cluster randomized controlled trial was conducted. All adolescents in the 8^th^ grade were eligible to participate, unless they, or their parents, declined their participation. Schools were the unit of randomization. Missing data were imputed and intent to treat analyses were conducted. The results were analyzed using Latent Growth Curve Modeling across the 24-months follow-up period.

**Results:**

The total sample consisted of 1341 adolescents (*M*age = 13.91, *SD* =0.55, 47.3% girls, 83.1% Dutch ethnicity). The intervention and control condition consisted of 634 adolescents from 4 schools and 707 adolescents from 5 schools, respectively. OVK did not have an effect on depression, anxiety, hopelessness, happiness, and life satisfaction, but promoted cognitive coping over the course of the follow-up period. OVK showed small iatrogenic effects on optimism, active coping, social self-efficacy and school grades directly post intervention, but these effects disappeared during the follow-up period. Finally, none of the outcome variables moderated the intervention effect on depressive symptoms.

**Conclusions:**

The universal resiliency training OVK was not effective in this Dutch sample. Implications for research and practice were discussed.

**Trial registration number:**

NTR2879

## Background

The percentage of adolescents who experience elevated levels of depressive symptoms increases across adolescence from the age of 13, with girls showing a higher increase compared to boys [1, 2]. During this period, the one year point prevalence of a depression disorder increases as well [3], and the lifetime prevalence rises from 8.4% at age 13-14 to 15.4% at age 17-18 [4]. Symptoms of depression negatively influence social, peer, and family functioning [5, 6], and they are associated with future depression disorder [7, 8]. Considering the increase in prevalence and the detrimental consequences, the prevention of depressive symptoms is important.

Depression prevention programs directed at indicated and targeted population are more likely to be effective in preventing depressive symptoms and more likely to yield in larger effect sizes compared to universal approaches (for reviews see [9–11]). However, a recent review indicated that universal depression prevention could also be effective in preventing depressive symptoms [12]. Effective depression prevention programs are often based on Cognitive Behavioral Therapy (CBT). The need to investigate moderators of prevention effectiveness has been emphasized [13], since we have only sparse information on who achieves the best results from the CBT depression prevention programs. In addition, it is relatively unclear whether CBT intervention and prevention programs affect the proposed mechanisms of change in CBT, such as cognitive vulnerabilities, coping skills, and self-efficacy [14].

An example of an effective depression prevention program for indicated and targeted populations is the Penn Resiliency Program (PRP). PRP has shown to reduce depressive symptoms [15], to reduce anxiety symptoms and feelings of hopelessness, and to promote active coping in targeted populations [16]. Besides, in participants at high risk for depression, PRP promoted adaptive explanatory style for positive events, and prevented depression, anxiety and adjustment disorders combined [17]. PRP has shown to be less effective in universal trials and when delivered by other professionals rather than the research team members [15]. However, it has been found that PRP reduces depressive symptoms at post intervention only, implemented as universal prevention and evaluated in a non-randomized trail [18].

Despite these promising results, the Dutch equivalent of PRP, Op Volle Kracht (OVK), has not been found to be effective in preventing depressive symptoms at a two-year follow-up implemented, either as a universal prevention [Tak YR, Lichtwarck-Aschoff A, Gillham JE, Van Zundert RMP, Engels RCME: **Universal school-based depression prevention ‘Op Volle Kracht’: A longitudinal cluster randomized controlled trial**, Submitted], or at one year follow-up implemented as a selective depression prevention [19]. In addition, adolescent baseline depressive symptom level and gender did not moderate universal prevention effectiveness in these trials. In contrast, a short version of OVK implemented as indicated prevention in girls only groups reduced depressive symptoms in adolescent girls at six months follow-up [20].

The current paper assessed possible moderators of universal depression prevention effectiveness, analyzing whether baseline levels of anxiety, hopelessness, optimism, happiness, life satisfaction, coping or self-efficacy moderated the effectiveness of OVK in preventing depressive symptoms. In addition, the study analyzed the longitudinal effectiveness of OVK in preventing anxiety symptoms and hopelessness, and in promoting optimism, happiness, life satisfaction, adaptive coping, and self-efficacy.

The OVK program is an adapted version of the validated Penn Resiliency Program (PRP) [15, 21]. The content, examples, layout and structure of the program were adapted to suit the needs of Dutch adolescents [22]. In the OVK program, attention is directed at becoming aware of one’s thoughts, to recognize negative thoughts, to challenge those thoughts, and to change these negative thoughts into more realistic and optimistic ones. In addition, adolescents learn to cope with their problems and to improve their social skills and problem solving. Both programs are based on CBT [23], the ABC-model [24], and the hopelessness theory of depression [25]; as is described more elaborately in previous papers on OVK [Tak YR, Lichtwarck-Aschoff A, Gillham JE, Van Zundert RMP, Engels RCME: **Universal school-based depression prevention 'Op Volle Kracht': A longitudinal cluster randomized controlled trial**, Submitted, [22].

With regard to moderators of the effectiveness of CBT depression prevention, researchers have described that age and depressive symptom levels at baseline are positively associated with prevention effects [11]. In addition, only adolescents reporting high baseline levels of hopelessness showed a decrease in depressive symptoms after completing the prevention program [16], and only adolescents reporting elevated levels of anxiety showed a reduction in depressive symptoms up to 12-months follow-up [26]. Therefore, we hypothesized that OVK will be effective in preventing depressive symptoms among adolescents displaying high baseline levels of anxiety and hopelessness.

Studies that assessed the proposed mechanisms of change of CBT prevention programs have shown contrasting results. It has been found that CBT treatments effectively promote cognitive change in adolescents displaying depression disorder but do not influence coping in adolescents [14]. In contrast, CBT programs implemented as universal and selective prevention and intervention did not affect attributional style or self-esteem, but successfully reduced hopelessness in adolescents [27]. Few studies have tested whether CBT prevention promotes positive outcomes, such as optimism, life satisfaction, and happiness during adolescence. This is important, since adults and adolescents who are more optimistic are more resilient, more satisfied with life [28, 29], and less vulnerable to depression [30]. Besides, higher levels of self-efficacy among adolescents [31] and the use of coping strategies as problem solving and reappraisal are associated with fewer depressive and anxiety symptoms in adults and adolescents [32]. It has been found that CBT implemented as indicated prevention promoted optimistic explanatory style as well as life satisfaction and happiness, and reduced depressive and anxiety symptoms in adolescents up to six months follow-up [33]. In addition, the improvements in explanatory style partly mediated these prevention effects. The current study therefore hypothesized that OVK will promote optimism, happiness, life-satisfaction, coping, and self-efficacy.

## Methods

### Trial design & randomization

To test the longitudinal effects of the OVK program, a two arm parallel cluster randomized controlled trial was performed with baseline, post, and follow-up assessments at six months, one year, 18 months and at two years [Tak YR, Lichtwarck-Aschoff A, Gillham JE, Van Zundert RMP, Engels RCME: **Universal school-based depression prevention 'Op Volle Kracht': A longitudinal cluster randomized controlled trial**, Submitted, [22]. Adolescents were the unit of analysis and to minimize contamination between research conditions, we randomized schools to intervention or control condition (lessons as usual) with allocation ratio of 1:1. The randomization was stratified for educational level and was performed by an independent statistician. Schools and adolescents were not blind to condition. The ethics committee of the Faculty of Social Sciences at the Radboud University Nijmegen approved the research protocol and trial design (number 16122010) registered by the Dutch Trial Registration (NTR2879).

To detect a low to medium effect (Cohen’s d =0.20) at one year follow-up on a dichotomous outcome (CDI = > 13 yes/no) while considering 20% attrition over the two year follow-up period, the clustering of participants within schools, and loss of power due to multiple imputation, the study needed to recruit 662 participants per condition (alpha < .05, power = .80). The actual intervention group consisted of 4 schools and 655 adolescents of whom only .05% declined participation (3 out of 655). The actual control group consisted of 5 schools and 735 adolescents of whom only 1.4% (10 out of 735) declined to participate. For participant characteristics, see Table 1.

**Table 1:** **Baseline characteristics**

	Intervention condition	Control condition	Total	Difference
	n =634	n =707	N =1341	I-C
	***M***(***SD***)	***M***(***SD***)	***M***(***SD***)	***p***
Gender (%)				.601
Girls	47.5	47.1	47.3	
Boys	52.5	52.9	52.7	
Age	14.0 (0.5)	13.9 (0.6)	13.9 (0.6)	.045
Ethnicity (%)				.002
Dutch	79.0	86.8	83.1	
Other ethnicity	21.0	13.2	16.9	
School level (%)				.015
PVSE (Low)	11.4	3.1	7.0	
HGSE (Middle)	48.4	53.6	51.2	
PUE (High)	40.2	43.3	41.8	
Primary outcome				
Depressive symptoms	7.4 (5.6)	7.7 (5.8)	7.6 (5.7)	.430
Secondary outcomes				
Anxiety	6.6 (5.6)	6.9 (5.6)	6.8 (5.6)	.761
Hopelessness	3.5 (2.8)	3.6 (2.8)	3.5 (2.8)	.666
Happiness	7.9 (1.4)	7.8 (1.3)	7.8 (1.4)	.598
Life satisfaction	30.6 (7.2)	30.4 (6.8)	30.5 (7.0)	.675
Optimism	15.2 (4.2)	15.4 (4.0)	15.3 (4.1)	.008
Active coping	30.0 (7.2)	29.2 (6.9)	29.6 (7.1)	.570
Cognitive coping	27.6 (7.0)	27.0 (6.7)	27.3 (6.8)	.423
Distraction coping	17.8 (4.6)	17.5 (4.9)	17.7 (4.7)	.621
Avoidance coping	25.5 (5.7)	25.3 (5.6)	25.4 (5.7)	.544
Seeking support coping	18.7 (6.6)	18.3 (6.2)	18.5 (6.4)	.688
Academic self-efficacy	24.6 (5.3)	24.5 (5.2)	24.5 (5.3)	.776
Social self-efficacy	26.6 (4.3)	26.1 (4.3)	26.4 (4.3)	.299
Emotional self-efficacy	24.3 (5.2)	23.9 (5.1)	24.1 (5.2)	.316
Ancillary outcomes				
School grades	7.0 (1.1)	7.0 (1.1)	7.0 (1.1)	.638
Classroom atmosphere	4.0 (0.7)	3.9 (0.7)	4.0 (0.7)	.071
Alcohol use past 4-weeks %	21.0	21.2	21.1	.975
Current smoking %	5.4	6.1	5.8	.433
Truancy %	4.4	4.4	4.4	.889

*Note*. Logistic regression analyses were used to calculate differences between I – C. I = intervention group, C = control group. PVSE = pre-vocational secondary education (Dutch translation is VMBO), HGSE = higher general secondary education (HAVO), PUE = pre-university education (VWO). Optimism was not significantly different between conditions when tested separately (*OR* =0.93, CI 95%: 0.79 – 1.08, *p* = .337).

### Procedure & participants

All adolescents in 8^th^ grade of secondary schools preparing for vocational up to university level were eligible to participate. The passive consent procedure was used, but parents were free to withdraw their adolescent from the study at any time if the adolescent did not want to participate or if they did not want their son/daughter to participate. Study outcomes were assessed by administering 50-minute questionnaires to adolescents during school hours in the classroom setting. Adolescents who were not present at school were asked by e-mail or mail and subsequently by phone to complete the questionnaire at home. Adolescents who completed the questionnaires at their leisure time at home received a gift voucher of €7.50 per assessment. At one year follow-up, five additional gift vouchers of €20 were allotted for adolescents who were absent during the assessment at school to increase their participation rate. At the two-year follow-up, all participants who completed the assessment received a gift voucher of €7.50.

### Intervention

Adolescents in the intervention condition received the program OVK consisting of 16 weekly 50-minute lessons offered during the ‘mentor lesson’ from February 2011 until June 2011. During the mentor lesson, the mentor from each class discusses school related aspects, such as school activities and classroom atmosphere. In the intervention group, classrooms were split to create groups of 10-16 students. In some schools, teachers created these groups to ensure that the children who were most likely to cause trouble were evenly distributed across the OVK intervention groups. The control condition received lessons as usual. The OVK prevention program is based on the Penn Resiliency Program [21]. Adaptations in content, structure, and layout were made to match the interests and culture of the Dutch adolescent population. The first part of the program covers CBT principles and the second part focuses on coping, decision-making and problem solving. During the lessons, adolescents received classical instruction, and practiced the skills by means of role-plays, skits, and discussions. Subsequently, they completed pen and paper assignments in their personal OVK workbook. To foster the internalization and generalization of the skills learned, every lesson included homework assignments. The program included a two-hour booster session, which was delivered after the assessment at six months. During the booster session, adolescents rehearsed the main principles of OVK by watching a music performance about resiliency and by writing a poem about what they learned from OVK (see [22], for a detailed program description). Ten psychologists (1 per group) with varying experience in Cognitive Behavioral Therapy and teaching administered the OVK. One trainer was the principal author of this paper. All OVK group trainers completed a five-day OVK training in advance. Two experienced psychologists who were trained by members of the Penn Resiliency Team provided the OVK training. The number of OVK groups per trainer ranged from five to nine (*M* =5.67, *SD* =1.40). Program fidelity was monitored by organizing two meetings for group trainers with the OVK developers and research staff. The program fidelity was measured using self-reports, which were completed by group trainers after every OVK lesson.

### Measures

Two independent researchers translated the scales for anxiety, hopelessness, optimism, life satisfaction, happiness and self-efficacy into Dutch; subsequently, they discussed and solved any translation disagreements.

### Depressive symptoms

The primary outcome is the level of depressive symptoms measured with the Dutch version of the Children’s Depression Inventory (CDI) [34, 35], which has been shown to be reliable and valid [36]. Depressive symptoms were measured six times, at baseline and every follow-up assessment, up to two years follow-up. For each of the 27 items, adolescents had to indicate, which of the three statements best describes how they felt in the past two weeks. For example, ‘I am sad sometimes’ =0, ‘I am often sad’ =1, and ‘I am sad all the time’ =2. Depressive symptoms sum scores ranged from 0 – 54. The reliability of all assessments was high, with Cronbach’s alpha ranging from .84 – .91. Due to ethical considerations, item nine, which measures suicidal thoughts and ideation, was omitted from the questionnaire after the baseline assessment. To facilitate comparison with other studies, the sum scores were adjusted after omitting item nine by multiplying the mean item score times 27.

### Anxiety

Adolescents’ anxiety at baseline up to two years follow-up was assessed by the Revised Children’s Manifest Anxiety Scale (RCMAS), which has been found to be reliable and valid [37–39]. Adolescents had to indicate whether they agreed, yes =1, no =0, with 28 items included in the scale. Examples of anxiety items are, ‘I am afraid of a lot of things’, or ‘I am nervous’. The anxiety scores ranged from 0 – 28. Reliability was good to excellent on all assessments, with Cronbach’s alpha ranging from .88 – .92.

### Hopelessness

Hopelessness at baseline up to two years follow-up was assessed with the Beck Hopelessness Scale (BHS), which has shown to have good reliability and validity [40, 41]. The scale consists of 20 items, for example: ‘I look forward to the future with hope and enthusiasm’. Adolescents had to indicate whether the item was ‘true’ =1 for them or ‘not true’ =0. The hopelessness scores ranged from 0 – 20. Acceptable to good levels of reliability were obtained on all assessments, with Cronbach’s alpha ranging from .73 – .81.

### Happiness

Adolescents’ level of happiness at baseline up to two years follow-up was measured with the Cantril Ladder [42]. Adolescents were asked to mark the number that best corresponds with how they felt about their life at that moment. The ladder ranged from 0 = ‘very unhappy’, to 10 = ‘very happy’. This one item measure has been found to be reliable and valid [43].

### Life satisfaction

Life satisfaction at baseline up to two years follow-up was measured with the Students Life Satisfaction Scale (SLSS) [44]. This is a reliable and valid measure [45], and consists of seven items measured on a 6-point Likert-scale ranging from ‘strongly disagree’ =1, ‘to strongly agree’ =6. The sample items are: ‘My life is going well’ and ‘I have what I want in life’. Life satisfaction scores ranged from 7 – 42. Cronbach’s alpha ranged from .86 – .88, which indicates good reliability across the study.

### Optimism

The level of optimism at baseline up to two years follow-up was assessed by the Life-Orientation Test Revised (LOT-R), which is a reliable and valid measure [46]. The scale consists of six optimism items and four filler items measured on a 5-point Likert-scale, ‘strongly disagree’ =0, ‘to strongly agree’ =4. An example item is, ‘In uncertain times, I usually expect the best’. Optimism scores ranged from 0 – 24. Reliability was acceptable on all assessments, with Cronbach’s alpha ranging from .63 – .75.

### Coping

Adolescents’ coping at baseline up to 18 months follow-up was measured with the Dutch version of the Children Coping Strategies Checklist-Revised (CCSC-R), which is a reliable and valid measure [47]. Adolescents had to respond to 54 statements that all started with, ‘If I have a problem, I…’, followed by different scenarios, such as ‘Tell others how I like to solve the problem’. Adolescents had to indicate how often they responded in this way and choose one of the 4 responses, ‘almost never’ =1, ‘sometimes’ =2, ‘often’ =3, ‘almost always’ =4. The questionnaire consists of five subscales, active coping (12 items), for example, ‘Do something to make things better’; cognitive coping (12 items), for example, ‘Tell yourself that you know what to do’; distraction coping (nine items), for example, ‘Play sports’; avoidance coping (12 items), for example, ‘Just forget about it’; and seeking support coping (nine items), for example, ‘Tell others how you feel about the problem’. All coping scales achieved good reliability on all assessments. Cronbach’s alpha for active coping ranged from .88 –.91. Cronbach’s alpha for cognitive coping ranged from .87 – .91. Cronbach’s alpha for distraction coping ranged from .74 – .79. Cronbach’s alpha for avoidance coping ranged from .73 – .85. Cronbach’s alpha for support seeking coping ranged from .91 – .93.

### Self-efficacy

Academic, social, and emotional self-efficacy was assessed with the Self-Efficacy Questionnaire (SEQ) [48] at baseline up to the 18 months follow-up. The measure has been found to be reliable and valid. This questionnaire consists of three subscales: academic, social, and emotional self-efficacy, each measured with seven items. Adolescents had to indicate on each item how well they thought they could perform the task or the skill on a 5-point Likert scale ranging from 1 = not at all to 5 = very well. Subscale sum scores ranged from 7 – 35. An example item assessing academic self-efficacy is, ‘How well do you succeed in finishing all your homework every day?’ Cronbach’s alpha ranged from .86 – .90. A sample item of social self-efficacy is, ‘How well do you succeed in staying friends with other children?’ Cronbach’s alpha ranged from .79 – .91. A sample item of emotional self-efficacy is, ‘How well do you succeed in becoming calm again when you are very scared?’ The subscale showed good reliability on all assessments, with Cronbach’s alpha ranging from .85 – .91.

### School grades

Adolescents had to report the last grade they obtained on Dutch, English, Mathematics, Environmental Studies, Economics and Biology tests. Since not all adolescents took all courses, the mean grade was calculated for the subjects that they took. Mean grades ranged from 0 – 10.

### Classroom atmosphere

Three items were constructed to assess the relationship with classmates: ‘My classmates like to be together’, ‘My classmates are friendly and helpful’, ‘My classmates accept me’. Adolescents could answer on a 5-point liker scale ranging from 1 = strongly disagree to 5 = to strongly agree. Subscale sum scores ranged from 5 – 15. The scale was reliable, with Cronbach’s Alpha ranging from .76 – .90 across assessments.

### Alcohol consumption

Adolescents had to indicate how many alcoholic beverages they drank during the past four weeks. Since the variable showed a largely skewed distribution and transformations did not correct the skewness, the answers were dichotomized: I drank alcohol in the past four weeks, 1 = yes, 0 = no.

### Current smoking

Adolescents reported whether they currently smoked: Yes =1, no =0.

### Truancy

Adolescents reported the number of lessons they skipped. Since this variable had a largely skewed distribution and transforming the data did not improve the skewness, the data was dichotomized: Truancy yes =1, truancy no =0.

### Strategy of analyses

Differences at baseline between intervention and control condition in gender, age, school level, ethnicity, and outcome variables were assessed with logistic regression analysis and controlled for in subsequent analyses. Predictors of attrition across follow-up assessments were estimated with logistic regression analysis. For educational level, two dummy variables were created: Lower education consisted of pre-vocational secondary education (PVSE) =1 (which is in Dutch: VMBO) versus higher general secondary education (HGSE) =0 (HAVO), and pre-university education (PUE) =0 (VWO) [49]. Middle education consisted of HGSE =1 versus PVSE =0, and PUE =0.

The longitudinal effectiveness of OVK was analyzed based on the intent-to-treat framework (N =1,341). Missing data were imputed 20 times, auxiliary variables were included in the models, and the imputation was done separately for the control and intervention groups. These decisions were made since imputing the model several times and including auxiliary variables leads to more accurate standard error estimates [50]. Imputations were done by multiple imputation using the predictive mean matching method in SPSS 19. The intra-class correlation coefficient (ICC) for all outcome measures was calculated and ranged from .00 to .14 with a mean of .02, which implies that 2% of the variance in the outcomes was explained by school-related aspects. Therefore, all LGCM analyses controlled for the clustering of participants in schools.

To test the longitudinal effectiveness of OVK on depressive symptoms and secondary and ancillary outcomes, Latent Growth Curve Modeling (LGCM) was performed using Mplus [51]. First, single growth curves were assessed from post assessment up to 18 months or two years follow-up, depending on the number of follow-up assessments available for the outcome variable. This was done by estimating the post assessment level (intercept) and change over the follow-up period (slope) for all outcomes. Second, the model was assessed including study condition as predictor as well as the control variables age, ethnicity, school level, gender, and the baseline level of the outcome variable. Linear and quadratic slopes were tested, but since the variance of the quadratic slope was not significant for any of the outcomes, the quadratic slope was not included in the models. The model was considered to have a good fit when the fit indices were RMSEA < .05, CFI > .90. Since the chi-square value is less reliable with large sample sizes, it was not reported [52]. The analyses aimed to assess whether the secondary outcomes moderated the relation between the preventive effect of OVK and depressive symptoms over the two-year follow-up period. In these analyses, we controlled for multiple testing by applying a Bonferroni correction. The analyses for the secondary and ancillary outcomes were exploratory. Therefore, to minimize the risk on a Type II error, no Bonferroni correction was applied.

## Results

**Figure 1:**
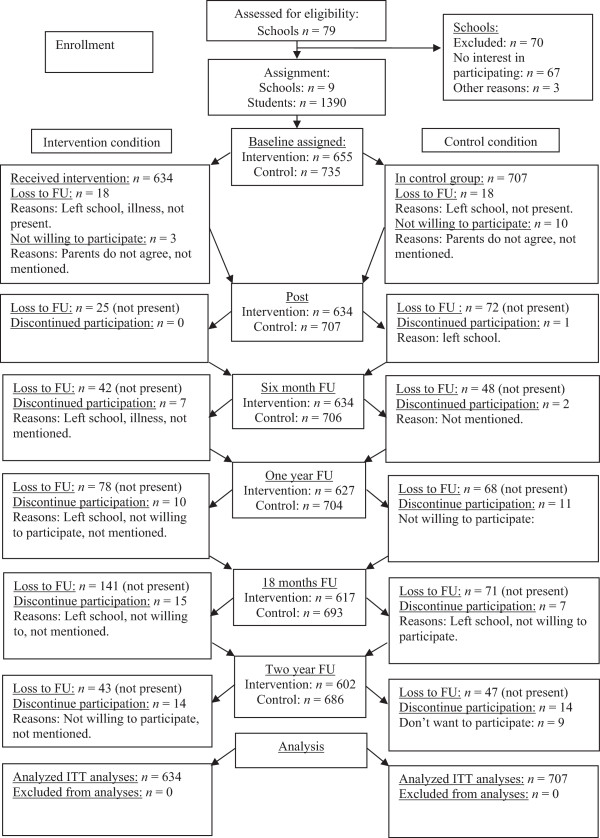
**Participant flow.**

From the 79 schools that were approached to participate in this project, 12 schools showed initial interest, and nine schools eventually agreed to participate. Four schools were allocated to the intervention condition (655 adolescents) and five schools to the control condition (735 adolescents). The intervention group consisted of 51 OVK groups of 10-16 students (Mode =12). Only 1.0% (13 of 1390) of the potential participants declined participation at baseline, and an additional 2.6% (36 of 1390) did not complete the baseline assessment for various reasons (see Figure 1 for the flow diagram). Attrition rates were low; the percentage of participants completing the questionnaire at baseline, post, six months, one year, 18 months, and two-year follow-up were 96.5%, 89.4%, 89.3%, 83.7%, 77.4%, and 84.5%, respectively. Overall, 1341 participants who completed baseline assessments, 634 from the intervention condition and 707 from the control condition, were included in the analyses.

Baseline characteristics are presented in Table 1. Some variables were distributed unevenly across conditions and were controlled for in subsequent analyses: PVSE versus HGSE and PUE (*OR* =0.31, 95% CI =0.18 - 0.52, *p* = .000), age (*OR* =1.24, 95% CI =1.01 - 1.53, *p* = .045), and ethnicity (*OR* =0.62, 95% CI =0.46 - 0.83, *p* = .002). Attrition analyses showed that across all follow-up assessments, girls (*OR* =0.50, 95% CI =0.30 - 0.83, *p* = .007) and adolescents in the control condition (*OR* =0.59, 95% CI =0.36 - 0.96, *p* = .033) were more likely to decline further participation. In addition, adolescents in PVSE were less likely to decline further study participation compared to HGSE and PUE (*OR* =0.19, 95% CI =0.07 - 0.51, *p* = .001). Moreover, adolescents in HGSE were less likely to decline further study participation compared to PVSE and PUE (*OR* =0.19, 95% CI =0.01 - 0.38, *p* = .000).

The OVK prevention was delivered according to the manual, since the treatment fidelity was 80%, according to the group trainers. Adolescents participated on average in 14-15 out of 16 lessons, and 67.8% (n =430) of the intervention group attended the booster session that was provided after the assessment at six months.

### Moderation of secondary outcomes on OVK effectiveness on depressive symptoms

OVK was not effective in preventing depressive symptoms (Table 2). For a more elaborative discussion about the effectiveness of OVK on depressive symptoms, see [Tak YR, Lichtwarck-Aschoff A, Gillham JE, Van Zundert RMP, Engels RCME: **Universal school-based depression prevention 'Op Volle Kracht': A longitudinal cluster randomized controlled trial**, Submitted]. Moderation analyses revealed that none of the secondary outcome variables, anxiety, hopelessness, happiness, life satisfaction, optimism, coping, self-efficacy, moderated the effect of OVK on depressive symptoms. A Bonferroni correction was applied since 13 tests were performed. Alpha’s were considered significant if smaller than .05 / 13 = .0038.

**Table 2:** **Standardized estimates of predictors, and control variables, and moderation of condition for the intercept and slope of depressive symptoms across the two-year follow-up period**

	Intercept	Slope		
	β (p-value)	β (p-value)	RMSEA	CFI
Control variables				
Age	0.077 (.004)	-0.115 (.000)		
Ethnicity	0.011 (.587)	0.021 (.787)		
Middle education	-0.065 (.047)	0.164 (.022)		
Low education	0.017 (.759)	0.103 (.548)		
Gender	0.073 (.077)	0.043 (.619)		
Depressive symptoms baseline	0.751 (.000)	-0.217 (.000)		
Condition	0.020 (.642)	-0.011 (.919)	.049	.903
Moderation secondary outcomes				
Anxiety	0.244 (.000)	-0.033 (.699)		
Hopelessness	0.046 (.387)	0.095 (.140)		
Happiness	-0.086 (.130)	0.036 (.817)		
Life satisfaction	-0.110 (.030)	-0.038 (.828)		
Optimism	-0.089 (.056)	-0.022 (.771)		
Active coping	-0.047 (.302)	0.039 (.585)		
Cognitive coping	-0.087 (.001)	0.003 (.961)		
Distraction coping	-0.018 (.373)	-0.045 (.512)		
Avoidance coping	-0.009 (.763)	0.015 (.788)		
Seeking support coping	-0.030 (.381)	0.054 (.358)		
Academic self-efficacy	-0.119 (.002)	0.035 (.588)		
Social self-efficacy	-0.012 (.755)	-0.056 (.598)		
Emotional self-efficacy	-0.053 (.289)	-0.039 (.624)		
Condition X anxiety	-0.047 (.277)	0.007 (.941)	.050	.900
Condition X hopelessness	0.011 (.773)	-0.032 (.633)	.050	.898
Condition X happiness	0.043 (.314)	-0.013 (.907)	.047	.905
Condition X life satisfaction	0.025 (.605)	0.015 (.889)	.046	.907
Condition X optimism	-0.008 (.824)	0.039 (.565)	.048	.906
Condition X active coping	-0.002 (.958)	-0.029 (.667)	.049	.903
Condition X cognitive coping	0.027 (.376)	0.019 (.768)	.048	.907
Condition X distraction coping	-0.004 (.864)	0.083 (.145)	.047	.908
Condition X avoidance coping	0.035 (.385)	0.065 (.156)	.047	.907
Condition X seeking support coping	-0.015 (.688)	-0.046 (.419)	.048	.906
Condition X academic self-efficacy	0.014 (.680)	-0.066 (.320)	.047	.908
Condition X social self-efficacy	-0.059 (.171)	0.036 (.635)	.047	.905
Condition X emotional self-efficacy	-0.026 (.457)	-0.002 (.976)	.047	.907

*Note.* Gender: female =0, male =1, Ethnicity: Dutch =0, not Dutch =1. Condition: 0 = control group, 1 = intervention group. Middle education: HGSE =1, PVSE and PUE =0. Lower education: PVSE =1, HGSE and PUE =0. Since 13 tests were performed, a Bonferroni correction was applied: alpha = .05 / 13 = .0038.

### Latent growth curve model findings for primary, secondary, and ancillary outcomes

First, the models for depression (primary outcome), anxiety, hopelessness, happiness, life satisfaction, optimism, coping, self-efficacy (secondary outcomes), grades, classroom atmosphere, alcohol use, smoking, and truancy (ancillary outcomes) were tested without the predictor and control variables in Model 1. For all outcome variables, model 1 had a reasonable to good fit, as can be seen in Table 3. The variance of the intercept was significant for all outcomes, indicating individual differences at post-assessment on the outcome variables. For avoidance coping, alcohol use during the past 4-weeks, and current smoking, the variance of the slope was not significant, indicating no differences among participants in the rate of change on these variables across the follow-up period. Therefore, LGCM was not conducted for these outcomes.

**Table 3:** **Model 1 for depressive symptoms, secondary outcomes, and ancillary outcomes**

		S (***p***)	Var (S) (***p***)	Var (I) (***p***)	RMSEA	CFI
PO	Depressive symptoms	.005 (.341)	.002 (.000)	.032 (.000)	.059	.892
SO	Anxiety symptoms	.003 (.145)	.001 (.010)	.027 (.000)	.032	.968
	Hopelessness	.007 (.044)	.001 (.000)	.014 (.000)	.044	.993
	Happiness	-.018 (.210)	.072 (.000)	1.074 (.000)	.034	.973
	Life satisfaction	-.021 (.140)	.025 (.000)	.531 (.000)	.044	.989
	Optimism	.015 (.178)	.013 (.000)	.212 (.000)	.058	.961
	Active coping	-.016 (.357)	.011 (.004)	.186 (.000)	.057	.962
	Cognitive coping	-.009 (.416)	.016 (.001)	.204 (.000)	.067	.948
	Distraction coping	-.014 (.114)	.007 (.004)	.156 (.000)	.069	.949
	Avoidance coping	-.005 (.415)	.006 (.061)	.115 (.000)	.060	.954
	Seeking support coping	.014 (.194)	.018 (.000)	.318 (.000)	.044	.983
	Academic self-efficacy	-.060 (.000)	.018 (.020)	.285 (.000)	.043	.942
	Social self-efficacy	-.041 (.064)	.025 (.006)	.237 (.000)	.053	.947
	Emotional self-efficacy	-.041 (.002)	.026 (.008)	.278 (.000)	.031	.970
AO	School grades	-.081 (.001)	.035 (.000)	.611 (.000)	.050	.771
	Classroom atmosphere	.010 (.714)	.020 (.000)	.288 (.000)	.039	.978
	Alcohol use past 4-weeks	.353 (.005)	.080 (.359)	.734 (.000)	.036	.984
	Current smoking	-.038 (.896)	.160 (.605)	.582 (.001)	.032	.989
	Truancy	.205 (.017)	.029 (.013)	.559 (.000)	.010	.998

*Note*. For dichotomous outcomes the WLSMV estimator was used, and unstandardized regression coefficients were provided, since Mplus does not provide standardized values for imputed data. S = slope, I = intercept, var = variance, PO = primary outcome, SO = secondary outcomes, AO = ancillary outcomes.

Second, for the primary, secondary, and ancillary outcomes, Model 2 included study condition as predictor and age, ethnicity, school level, gender, and the baseline level of the outcome variable as control variables. As can be seen in Table 4, OVK condition showed an iatrogenic effect on the intercept (the post assessment) of optimism, active coping, social self-efficacy, and school grades. This indicates that at post-assessment (immediately following the intervention), adolescents in the intervention condition reported less optimism (intervention group: *M* =14.65, *SD* =3.90; control group: *M* =15.80, *SD* =4.07; Cohen’s d = .29), less active coping (intervention group: *M* =28.87, *SD* =7.37; control group: *M* =30.23, *SD* =6.94; Cohen’s d = .19), less social self-efficacy (intervention group: *M* =25.63, *SD* =5.67; control group: *M* =26.58, *SD* =4.21; Cohen’s d = .19), and lower school grades (intervention group: *M* =7.07, *SD* =1.70; control group: *M* =7.22, *SD* =1.40; Cohen’s d = .09). In contrast, OVK condition was positively associated with the slope of active coping and social self-efficacy, indicating that from post assessment to 18 months follow-up, the iatrogenic effect of active coping and social self-efficacy diminished, since the intervention group showed a larger increase compared to the control group during this period. By means of separate regression analyses in which the control variables were included, it was analyzed whether condition predicted the final assessment. This showed that at 18 months follow-up, the intervention group did not differ in active coping (*β* =0.036, *SE* =0.045, *p* = .415; Cohen’s d = .11; intervention group: *M* =29.69, *SD* =6.67; control group: *M* =28.95, *SD* =6.89) or on social self-efficacy (*β* =0.060, *SE* =0.052, *p* = .253; Cohen’s d = .12; intervention group: *M* =25.78, *SD* =5.05; control group: *M* =25.17, *SD* =5.33) compared to the control group.

**Table 4:** **Model 2: Standardized estimates of OVK condition for the intercept and slope of secondary and ancillary outcomes across the two-year follow-up period**

		Intercept	Slope		
		β ***(p-value)***	β ***(p-value)***	RMSEA	CFI
Condition as predictor					
SO	Anxiety	-0.028 (.436)	0.079 (.303)	.037	.962
	Hopelessness	0.088 (.068)	-0.008 (.947)	.042	.952
	Happiness	0.070 (.089)	-0.026 (.599)	.032	.971
	Life satisfaction	0.038 (.238)	-0.044 (.569)	.035	.980
	Optimism	**-0.098 (.005)**	0.024 (.765)	.047	.954
	Active coping	**-0.144 (.002)**	**0.257 (.010)**	.049	.955
	Cognitive coping	-0.001 (.975)	**0.120 (.042)**	.044	.969
	Distraction coping	0.078 (.077)	-0.021 (.828)	.047	.953
	Avoidance coping				
	Seeking support coping	-0.026 (.545)	0.061 (.415)	.037	.980
	Academic self-efficacy	-0.022 (.367)	0.101 (.194)	.040	.952
	Social self-efficacy	**-0.135 (.000)**	**0.229 (.039)**	.040	.960
	Emotional self-efficacy	-0.038 (.093)	0.067 (.288)	.035	.960
AO	School grades	**-0.175 (.003)**	.092 (.353)	.044	.843
	Classroom atmosphere	-0.026 (.861)	-.055 (.757)	.030	.959
	Alcohol use past 4-weeks				
	Current smoking				
	Truancy	-0.152 (.462)	-.022 (.879)	.014	.979

*Note.* Condition: 0 = control group, 1 = intervention group. SO = secondary outcomes, AO = ancillary outcomes. In these analyses, control variables, baseline levels of outcome variables, and school clustering was taken into account. For the dichotomous outcome ‘Truancy’, the WLSMV estimator was used, and unstandardized regression coefficients were provided. Significant effects are presented in bold.

Although OVK condition was not associated with the slope of school grades or optimism across follow-up, adolescents did not differ significantly in school grades at two-year follow-up (*β* = -0.068, *SE* =0.043, *p* = .112; Cohen’s d = .17; intervention group: *M* =6.56, *SD* =1.01; control group: *M* =6.73, *SD* =0.99) or in optimism at two-year follow-up (*β* = -0.076, *SE* =0.043, *p* = .078; Cohen’s d = .18; intervention group: *M* =15.40, *SD* =4.27; control group: *M* =16.14, *SD* =4.10) compared to the control group. In addition, OVK condition was positively associated with the slope of cognitive coping from post assessment up to 18 months follow-up, which indicated that OVK promoted the use of cognitive coping during the follow-up period. At 18 months follow-up, the intervention group reported significantly more cognitive coping compared to the control group (*β* =0.095, *SE* =0.031, *p* = .002; Cohen’s d = .23; intervention group: *M* =28.39, *SD* =6.50; control group: *M* =26.84, *SD* =7.04).

## Discussion

Overall, the OVK depression prevention and resiliency training tested in a universal adolescent sample was not effective in preventing depression, anxiety, and hopelessness and in promoting optimism, life satisfaction, happiness, adaptive coping and self-efficacy. Baseline levels of secondary outcomes did not moderate the OVK effectiveness in preventing depressive symptoms. It should be noted that immediately following OVK, the intervention group reported less optimism, less active coping, less social self-efficacy, and lower school grades, but these effect sizes were considered small or trivial, and these iatrogenic effects diminished over the follow-up period. Besides these iatrogenic effects, OVK had a small positive effect on cognitive coping, since adolescents in the intervention group reported a stronger increase in cognitive coping over the follow-up period, and reported significantly higher levels of cognitive coping at 18 months follow-up, compared to the control group.

Factors related to study design and characteristics of the OVK program might explain why OVK was not successful in preventing negative development and promoting positive development. In previous studies, the PRP program has never been tested in a strictly universal trial with a randomized design. As discussed in the primary outcome paper of OVK [Tak YR, Lichtwarck-Aschoff A, Gillham JE, Van Zundert RMP, Engels RCME: **Universal school-based depression prevention 'Op Volle Kracht': A longitudinal cluster randomized controlled trial**, Submitted], the universal trials in which a preventive effect of PRP on depressive symptoms is reported included less than 20% of the addressed population, included participants through active consent, and provided the program after school hours [17, 53, 54]. In the current trial, more than 96% of the addressed sample participated in the OVK program through passive consent, and OVK was provided as a regular lesson during school hours. Adolescents who chose to participate in a study in their leisure time might differ from adolescents in the current trial on various characteristics as motivation to participate, psychological burden and support from parents, although mean depressive symptom levels of adolescents in the current trial did not differ from the ones in these PRP trials.

Second, characteristics of the sample could partly explain why OVK was not effective as a prevention program. The adolescents in the current study did not actively chose to participate in the OVK program, and they reported on average few depressive and anxiety symptoms, low levels of hopelessness and low levels of maladaptive coping. Therefore, OVK might have made them aware of their own negative cognitions and shortcomings in coping, since OVK focuses on coping with stressful daily events and changing negative thoughts into realistic ones. This awareness might have made adolescents feel more insecure about their own capacities. This might have resulted in a small but negative effect on optimism, social self-efficacy, active coping strategies, and even lower grades. Some other universal studies have also reported iatrogenic effects of universal CBT-prevention on depressive symptoms [19, 55]. Although the effect sizes reported in the current study were small or trivial, and no differences were apparent at two years follow-up, these findings argue against implementing OVK as universal prevention or resiliency training.

Answering the question about whether universal depression prevention in general is an effective way to prevent depression is beyond the scope of this article. In a recent review by Merry et al. (2012), it was concluded that although the effect sizes are smaller compared to indicated and targeted programs, universal programs are effective in preventing depressive symptoms [12]. In this review, the analysis of the effectiveness of universal prevention also included trials in which only 13 – 53% of the addressed population participated [53, 56] and included trials, which were directed at adolescents at an increased risk of depression [57, 58]. Inclusion of these trials makes the statement that universal depression prevention is effective less convincing. However, some universal trials that include more than 70% of the addressed population are effective in preventing depressive symptoms [59, 60]. To conclude, evidence regarding the effectiveness of universal trials is inconsistent. Therefore, identifying the effective components and mechanisms of these effective universal programs, i.e., the processes they address, the ways in which they engage adolescents, and the way they include the environment, is still very important.

An important aim of the current study was to identify moderators of OVK effectiveness on depressive symptoms and to investigate whether OVK had an effect on the proposed mediators of change in CBT based depression prevention. In the current study, none of the secondary outcomes moderated the prevention effectiveness on depressive symptoms. However, considering the characteristics of this universal sample, we see that not only do all mean scores of the secondary outcomes fall within the healthy range, there is also very little variation in these scores. Only very few adolescents report high levels of anxiety or hopelessness, for example. This restricted range in the secondary outcome measures makes it difficult to find any potential role of moderating factors. Future studies should focus on and investigate potential moderators of prevention effectiveness in order to improve depression prevention.

With respect to the possible mechanisms of change in CBT prevention, adolescents in the OVK condition reported a larger increase in cognitive coping across 18-month follow-up period compared to the control group. The focus of OVK is on changing cognitions, that is, on replacing negative thoughts by those that are more realistic. This might explain why the effects were found for the cognitive coping and not for the other coping strategies, which is in line with several other studies see [14]. Besides cognitive coping, OVK did not affect change in other possible mediators. This general lack of effect might be explained by the fact that adolescents in our sample were not highly motivated to fully engage in the program and to incorporate the skills learned in their own daily lives. The adolescents mentioned that they thought that the program was too long. They indicated that they would have preferred to have more time to discuss their own negative experiences, and they wanted to discuss the positive situations they had experienced as well. Motivation and engagement could be enhanced by adapting the program to better suit the individual needs of the adolescents, such as using adolescents own daily hassles and positive experiences in all exercises, as was also mentioned by Gillham et al. (2006) [17]. It has been shown that a certain degree of flexibility in applying treatment modules – rather that rigid adherence to the program protocol – is associated with better outcomes in CBT based interventions [61].

### Strengths and limitations

The main strength of this study is its randomized controlled design executed in a large sample. Second, a high percentage of the universal sample addressed for participation actually participated at baseline, and a small dropout percentage across the follow-up assessments was obtained, which increases the validity of our study. Third, the effect of OVK on possible mediators of the effectiveness of CBT-based prevention was examined. Fourth, the current study tested whether other psychological constructs, i.e. anxiety, hopelessness and optimism moderate the OVK effectiveness on depressive symptoms.

Besides its strengths, this study has some limitations. First, the current study was not able to test the effectiveness of OVK on attributions or cognitions. Future studies should fill this gap, since in CBT-based programs, the aim is to change negative cognitions [23]. Second, the measure used for happiness consisted of a single item, which might be less reliable and valid in comparison with a multiple item questionnaire, although this measure was used reliably in previous research [43]. Third, the LOT-R that assessed optimism showed low but acceptable reliability in the current sample, and it has shown comparable levels of reliability in other studies [29, 62]. It seems that the LOT-R is measuring two separate constructs, optimism and pessimism, since both constructs explain unique variance in well-being and are often only moderately correlated [62]. This could explain the low overall reliability of the optimism scale. In light of this low reliability, the results should be confirmed before firm conclusions can be drawn. The fourth limitation is that a recall bias might have been present in the way school grades were assessed. Adolescents had to self-report their latest grades for each subject by recall. Future studies should use official grades provided by schools. Besides, the latest grade does not always reflect the average grade for a specific subject. Therefore, the results concerning school grades should be interpreted with caution. Using self-reports to assess alcohol, smoking, and truancy may be a limitation as well, since adolescents might feel reluctant to report using those substances or skipping a class. However, previous researchers have shown that self-report is a reliable and valid way to assess smoking [63] and alcohol use [64]. Moreover, adolescents were assured their answers would be handled confidentially. A final shortcoming of the present study is that the current sample included a smaller percentage of adolescents belonging to minority groups and adolescents with a low educational background than is present in the Dutch population. Therefore, generalizing the results of this study to the entire Dutch population is not possible.

## Conclusions

OVK was not effective in preventing depressive and anxiety symptoms or in preventing hopelessness and increasing optimism, happiness, life-satisfaction, and self-efficacy. OVK showed small iatrogenic short-term effects on optimism, active coping, social self-efficacy, and school grades, but these disappeared over time. In addition, OVK showed a small positive long-term effect on cognitive coping. None of the secondary outcome variables moderated the intervention effect on depressive symptoms. The importance of investigating the effectiveness of prevention programs before implementing them is highlighted. Based on this study, it can be concluded that OVK should not be implemented as universal prevention in its current form, although future research should affirm or attest these statements before firm conclusions can be drawn.
